# Transcriptomics and magnetic resonance imaging in major psychiatric disorders

**DOI:** 10.3389/fpsyt.2023.1185471

**Published:** 2023-06-09

**Authors:** Jing-Wen Fan, Yue-Wen Gu, Dong-Bao Wang, Xiao-Fan Liu, Shu-Wan Zhao, Xiao Li, Baojuan Li, Hong Yin, Wen-Jun Wu, Long-Biao Cui

**Affiliations:** ^1^Department of Clinical Psychology, Fourth Military Medical University, Xi'an, China; ^2^Department of Radiology, Xijing Hospital, Fourth Military Medical University, Xi'an, China; ^3^Schizophrenia Imaging Lab, Fourth Military Medical University, Xi’an, China; ^4^Department of Psychiatry, The First Affiliated Hospital of Chongqing Medical University, Chongqing, China; ^5^School of Biomedical Engineering, Fourth Military Medical University, Xi'an, China; ^6^Department of Radiology, Xi'an People's Hospital (Xi'an Fourth Hospital), Xi'an, China; ^7^Department of Psychiatry, Xijing Hospital, Fourth Military Medical University, Xi'an, China; ^8^Department of Radiology, The First Affiliated Hospital of Xi’an Jiaotong University, Xi’an, China

**Keywords:** MRI, transcriptomics, major depressive disorder, bipolar disorder, schizophrenia

## Abstract

Major psychiatric disorders create a significant public health burden, and mental disorders such as major depressive disorder, bipolar disorder, and schizophrenia are major contributors to the national disease burden. The search for biomarkers has been a leading endeavor in the field of biological psychiatry in recent decades. And the application of cross-scale and multi-omics approaches combining genes and imaging in major psychiatric studies has facilitated the elucidation of gene-related pathogenesis and the exploration of potential biomarkers. In this article, we summarize the results of using combined transcriptomics and magnetic resonance imaging to understand structural and functional brain changes associated with major psychiatric disorders in the last decade, demonstrating the neurobiological mechanisms of genetically related structural and functional brain alterations in multiple directions, and providing new avenues for the development of quantifiable objective biomarkers, as well as clinical diagnostic and prognostic indicators.

## Introduction

The recent Global Burden of Diseases, Injuries, and Risk Factors Study 2019 has shown that major psychiatric disorders remain among the top 10 leading causes of mental health (1). We selected a series of particularly interesting and important previous studies that have focused on major psychiatric disorders through a combination of transcriptomics and magnetic resonance imaging (MRI). Understanding the core transcriptomics-mapped neuroimaging phenotype of major psychiatric disorders, i.e., major depressive disorder (MDD), bipolar disorder (BD), and schizophrenia (SZ), is critical for diagnosis and treatment (2). Currently, we still lack a precise treatment strategy to effectively improve the situation.

Converging evidence increasingly suggests that psychiatric disorders have genetic heterogeneity and alterations in brain structure and function (3). However, the correlation and cross-validation between structural brain changes and gene expression are unclear (4). Recent years have provided new ideas based on quantitative analysis of the brain’s structural morphology and functional activity by structural and functional MRI (fMRI) techniques, combined with trans-scale and multi-omics approaches supported by transcriptomic data analysis (5, 6). The image-transcriptome analysis consists of three stages: processing transcriptional map data, linking expression measurement to neuroimaging phenotypes, and evaluating gene specificity and enrichment (6). Modern whole-brain transcriptional maps provide an unprecedented opportunity to study the molecular correlation of brain tissues. There are many publicly available human brain genetic databases that provide important data for neuroscience research, linking clinical data with genetic data, indicating specific gene expression and basic biochemical properties at each brain anatomical locus (7), most commonly Allen Human Brain Atlas. Scientists can use publicly available databases to understand how genes operate in the brain and further explore the neurobiological mechanisms of disease. A multi-omics and cross-scale approach allows for a clearer and more intuitive elucidation of the neuropathological and genetic mechanisms underlying major psychiatric disorders. The neuropathological and genetic mechanisms of major psychiatric disorders can be elucidated more clearly and intuitively, appropriate biomarkers of major psychiatric disorders etiology can be identified, and reliable evidence can be provided to improve the accuracy of major psychiatric disorders diagnosis, grasp disease progression, assess the effectiveness of treatments with psychotropic drugs and develop individualized clinical treatments.

## Transcriptomics and MRI in major depressive disorder

Major depressive disorder (MDD) is a common and complex mental illness with a high genetic correlation characterized by depressed moods, diminished interests, impaired cognitive function, and vegetative symptoms, such as disturbed sleep or appetite (8). Studies of MDD suggest a genetic basis of the illness that alters brain function and morphology. Researchers worldwide have also used neuroimaging technology to identify the diagnostic biomarkers for MDD.

Evolutionarily conserved genes and their related molecular pathways can serve as a bridge between human and mouse studies, expanding our understanding of biological pathways that mediate individual behavioral differences and psychopathological risks. Joeyen-Waldorf et al. (9) through comparative gene array analysis of serotonin transporter knockout mice (a genetic animal model replicating features of human depression) and transcriptomic data from postmortem brain tissue from MDD patients, found that 31 genes in the amygdala and 20 genes in the cingulate gyrus have consistent and significant changes in expression. In addition, they found the upregulation of the ADCY7 transcript in the amygdala by quantitative polymerase chain reaction, confirming that ADCY7 is related to MDD, and found that rs1064448 (one of the SNPs of the ADCY7 haplotype locus), in which T allele carriers had greater left amygdala reactivity than G allele homozygous carriers. These results suggest that ADCY7 plays a role in the molecular and neural mechanisms of regulating affect and mood regulation and in the pathophysiology of MDD (9).

Morphometric similarity networks (MSN) are a new approach to human cortical network mapping. MSN measures the interregional correlation of multiple macro- and micro-structural multimodal MRI variables in a single individual and differs from structural covariance analysis estimating the interregional correlation of a single macro-structural variable (like cortical thickness or volume) measured in multiple individuals. Li et al. (10) reported the link between whole-brain gene expression and changes in MSN in MDD relative to healthy controls. MSN analysis showed that compared with HC, MDD patients had lower MSN weights in the left upper frontal cortex and increased MSN weights in the left medial orbitofrontal cortex, isthmus of the cingulate cortex, lateral occipital cortex, and right lateral occipital cortex. Decreased MSN in the regions of MDD patients implies reduced morphological similarity between these regions and the rest of the cortex, and MDD patients have reproducible differences in cortical structure compared to controls. Using a human whole-brain transcriptomic dataset, 8 of 12 MDD-related genes were significantly associated with regional changes in MSN, including five negative correlations (i.e., CNR1, HTR1A, PDE1A, SST, and TAC1) and three positive correlations (i.e., ARRA2A, CHRM2, and CUX2). Analysis of cell type-specific signature genes suggests that changes in MSN in individuals with MDD were significantly enriched for biological processes associated with inflammation in microglial and neuronal cells (10).

Some circRNAs are highly expressed in the brain and are highly active in synapses involving nervous system development and differentiation, suggesting that circRNAs may be essential for understanding neuropsychiatric disorders (11). In an independent validation set, Shi et al. (12) verified the differential expression of circRNAs in healthy people and MDD patients. The results showed that compared with the control group, the level of circFKBP8 in MDD patients was significantly decreased, and the level of circMBNL1 was significantly increased. The circMBNL1 associates the HAMD-24 score through brain-derived neurotrophic factor (BDNF), and it is negatively correlated; the low-frequency fluctuation amplitude of the right orbital middle frontal gyrus is positively correlated with the expression of circFKBP8 and circMBNL1. The results of an interventional study on rTMS treatment showed that antidepressant treatment increased the expression of circFKBP8, and the expression of circFKBP8 was negatively correlated with HAMD-24 and Self-Rating Depression Scale scores. Whole blood circFKBP8 and circMBNL1 may be potential biomarkers for the diagnosis of MDD, and circFKBP8 may show great potential for prediction of the efficacy of antidepressant therapy (12).

The functional connectivity gradient provides us with a way to describe the brain connectome. If the functional connectivity patterns between voxels are more similar, the closer they are on the axes of the corresponding functional connectivity gradient. The different functional modules are arranged on the axes defined by these functional connectivity gradients according to their macroscopic functional hierarchies (13, 14). Xia et al. (15) reported for the first time the connectivity gradient dysfunction in patients with major depression and its association with transcriptional profiles. Global indicators of the primary-to-transmodal gradients (gradient explanation ratio, gradient range, and gradient variation) were found to be significantly lower in depressed patients than in healthy controls. Abnormalities in the patients’ local gradient scores were mainly located in the DMN, sensorimotor and visual cortices, involving both high-level abstract cognition and primary perceptual processing. Based on the public database of human gene expression profiles from the Allen Institute for Brain Science, the team used a combined brain connectome-transcriptome analysis to reveal that the abnormal pattern of local gradient scores was significantly associated with the expression of genes related to trans-synaptic signaling and calcium binding, elucidating the underlying molecular mechanisms of brain connectivity gradient disorders in depression (15).

There are large-scale epidemiological studies indicating that the lifetime prevalence of depression is higher in females than in males, and that sex differences in human brain development may be associated with sex differences in the incidence of depression during adolescence. It has been revealed that sex-differentiated development of functional connectivity is located in the DMN, ventral attentional, and limbic networks, as well as subcortical nuclei (16). Talishinsky et al. (17) found that depression was associated with both males and females in DMN with significant connectivity abnormalities, and the brain regions with the most significant gender-specific effects were the dorsomedial prefrontal cortex and subgenual cingulate cortex. The article prioritized at least six genes associated with depression: in women, TMEM161B-AS1 and KLHDC8B; in men, PRSS16, MRM2, ZKSCAN8P1, and PCDH8-a known regulator of activity-dependent synaptic reorganization. They may be involved in regulating connectivity changes in depression-related networks, and these findings have important implications for biomarker development and fMRI-guided therapeutic neuromodulation (17).

## Transcriptomics and MRI in bipolar disorder

BD is a serious mental disorder characterized by mania, depression, and neurocognitive impairment (18). Research on BD has shown that the genetic basis of BD alters brain function and morphology. However, it is unclear how BD-related genes affect brain structure and which brain regions are most important in BD.

McCarthy et al. (19) identified 58 genes potentially associated with BD and brain regions associated with BD through a genome-wide association study (GWAS) and classified 22 of these genes with anatomically distinct expression patterns into three gene expression clusters (C1: ATP6V1G3, GATA5, GPRB81, NGF, NPAS3, PAX1, SPERT, TNR, ZMIZ1; C2: ADCY2, CACNB3, DLG2, KDM5B, KIF1A, MAPK10, RIMBP2, UBE2E3, UBR1; C3: ANK3, STK39, FAM155A, SIPA1L2). Among these 22 genes, five genes, ATP6V1G3, ADCY2, CACNB3, RIMBP2, and UBR1, were significantly associated with BD. C1 is highly expressed in the parahippocampal gyrus, hippocampus, and posterior thalamus; C2 is highly expressed in the hippocampus, temporal cortex, and certain parts of the midbrain, but very low in the striatum and posterior thalamus; C3 is highly expressed in the posterior thalamus and very low expression in the caudate nucleus and putamen. After a meta-analysis of MRI results, it was found that the frontal cortex, cingulate cortex, insular cortex, temporal cortex, cerebellum, and parietal cortex had greater density/volume in the control group; in contrast, the density/volume of the left cingulate gyrus, left anterior hippocampus and left paracentral lobule was greater in BD patients. The brain regions with the highest and lowest expression of these genes did not strongly overlap with abnormal brain regions in BD patients, except the parahippocampal gyrus, inferior/superior temporal gyrus, and cerebellar earthworms. The findings of McCarthy et al. (19) suggest that expression profiles of BD-associated genes do not explain the majority of structural abnormalities observed in BD, but may be useful in identifying new candidate genes.

## Transcriptomics and MRI in schizophrenia

SZ is a severe and debilitating psychiatric disorder that is among the world’s top 10 causes of long-term disability. The symptoms of SZ include psychosis, apathy and withdrawal, and cognitive impairment, which lead to problems in social and occupational functioning, and self-care (20). However, the biological mechanism of SZ is still unclear. At present, the diagnosis of SZ mainly depends on the determination of patients’ symptoms, illness duration, dysfunction, and exclusions. Currently, many researchers focus on the correlation between neural connectivity and function, as well as polygenic models of inheritance in genetic research. The integration of multi-omics data has emerged as a way to provide a more comprehensive view of biological complexity, which is critical for translating clinical benefit into assessment and intervention in patients with SZ (21).

SZ is a disease associated with human brain evolution. The evolution of cognitive networks that promote advanced brain function may increase the risk of brain dysfunction. Wei et al. (22) found that compared with chimpanzees and macaques, human accelerating region (HAR) genes have different expression levels in human high-order cognitive networks. Genes with high expression in the DMN are involved in the formation of synapses and dendrites. The HAR/HAR-BRAIN gene was significantly associated with the genetic variation of SZ. The voxel-based morphometry study showed that the cortical expression pattern of the HAR-BRAIN gene significantly overlapped with that of cortical involvement in mental disorders. This suggests that genes related to human evolution may be associated with the occurrence and development of mental diseases, and lay the foundation for the biological mechanism of SZ in the future (22).

Working memory deficiency is one of the core symptoms of SZ, which is related to the abnormal activity of the prefrontal cortex. Some studies have shown that the dopaminergic dysfunction in the subcortical region and prefrontal cortex is the key pathophysiological mechanism of SZ. Pergola et al. (23) analyzed two different postmortem prefrontal mRNA datasets to identify the DRD2 co-expression pathway, which is rich in SZ risk genes, and discovered non-coding single nucleotide polymorphisms (SNPs) linked to co-expression. The polygenic co-expression index (PCI) was used to estimate the co-expression level of the DRD2 pathway in two separate health samples. They discovered that the higher the PCI, the more the DRD2 pathway was co-expressed in the prefrontal cortex, the more active the prefrontal cortex was, and the longer the working memory reaction time was. Patients with a higher PCI responded better to antipsychotic medication in two separate groups of SZ patients. The identification of this DRD2 co-expression pathway demonstrates that gene co-expression may filter SCZ risk genes into biological pathways that are linked to intermediate phenotypes and clinically relevant information (23).

**Figure 1:**
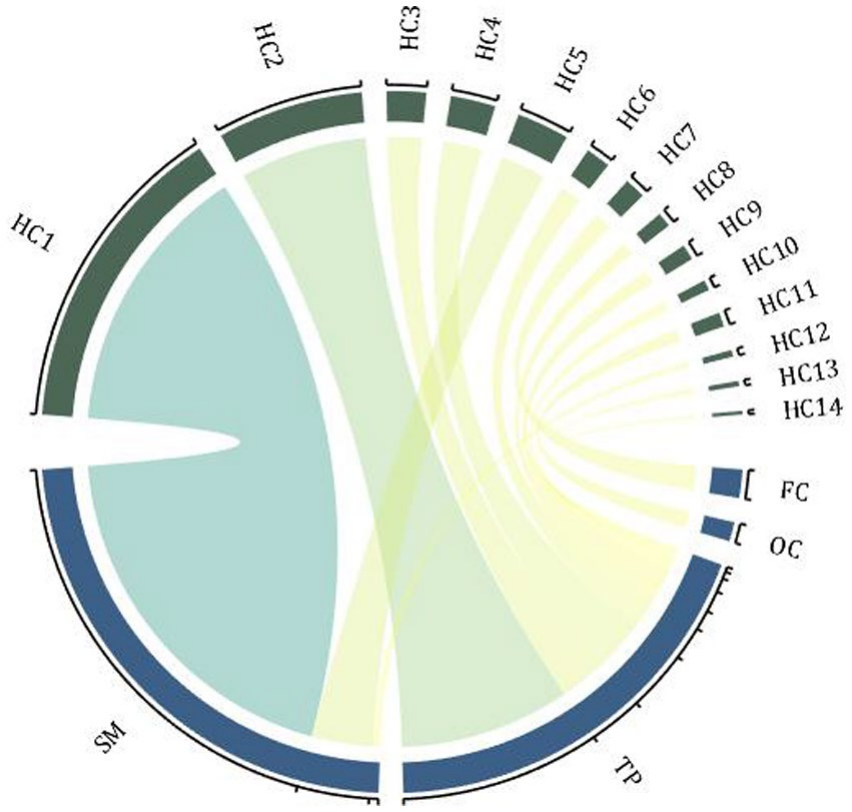
Hot clusters and associated genes. The enrichment distribution of 16 hot clusters in 4 brain regions, and the width of the arcs indicates the weight of the cluster size. FC, frontal cortex; TP, temporal and parietal regions; SM, sensory-motor regions; OC, occipital regions; HC, hot clusters (associated genes: HC1-CACNA1A, ERBB4; HC2-PPP1R1B; HC3-ATP2B2; HC4-PPP3CA, NRG3; HC5-ZBTB20, RELN; HC6-OPCML; HC7-INPP4B; HC8-ZNF365; HC9-ANK3; HC10-ZBTB20; HC11-PSAP; HC12-BMP6, EIF2B5; HC13-NRG3, PRKCA; HC14-FGF1).

Ma et al. (24) used multi-scale analysis of genomics, transcriptomics, neuroimaging, and clinical measurement data to explore the etiology of SZ. They found 19 different genes associated with 16 brain regions, mainly in the frontal cortex, sensory-motor areas, and temporal and parietal regions. Candidate genes distributed in the 16 AAL brain regions were classified into 16 hot clusters (see Figure 1 for the specific relationship between these genes and regions). Further subtype analysis was performed on patients according to the gray matter changes in the target brain area, and their symptoms were further characterized. It was found that patients with different subtypes had distinguishable severity of symptoms, that is, different brain structures had a distinguishable effect on SZ symptoms (24).

Transforming growth factor-beta 1 (TGF-β1) is a cytokine synthesized and secreted by astrocytes and microglia in the brain. TGF-β1 signaling has important implications for brain development and regenerative processes by regulating neurotransmission, synaptic remodeling, and brain connectivity (25). TGF-β1 may also be involved in the onset of SZ and cognitive deficits. Pan et al. (26) found that TGF-β1 was higher in patients with SZ than in controls at the mRNA level as well as at the protein level. Compared to controls, the cortical thicknes of the lateral occipital cortex in patients with SZ was significantly reduced and negatively correlated with the level of TGF-β1 protein. By correlating between plasma TGF-β1 levels and MCCB total and subscores, TGF-β1 levels were found to be negatively correlated with visual cognition in patients with SZ, and the lateral occipital cortex mediated the effect of TGF-β1 on visual cognition. TGF-β1 levels in peripheral blood may be a valid biomarker for detecting cortical or cognitive impairment in patients with early SZ (26).

Brain morphology differs significantly between patients with SZ, but the cellular and genetic basis for this heterogeneity is unclear. Di Biase et al. (27) measured the standard deviation range of 34 cortical regions in patients with SZ, and used gene sets data from Allen Brain Institute to mapping cell type-specific gene expression patterns in the human brain. Cell types included astrocytes, endothelial cells, oligodendrocyte progenitor cells (OPC), excitatory neurons and inhibitory neurons. They systematically combined the information on three biological scales —gene, gene expression and brain morphology to explain the mechanism of cortical thickness heterogeneity in patients with SZ. In the predominantly neuronal/endothelial subtype, cortical thickness thinning was associated with a higher polygenic risk of SZ. However, in the predominantly glial/OPC subtype, regional cortical thinning was weakly associated with neurons, astrocytes, and OPCs. This result suggests that cortical thickness heterogeneity in SZ is associated with inter-individual differences in cell type-specific function, contributing to an enhanced understanding of disease heterogeneity and paving the way for psychiatric investigation and intervention (27).

## Conclusion

Major psychiatric disorders are pervasive multifactorial disorders with various changes in brain function as well as brain structure and are hereditary. In SZ, structural brain changes are concentrated in the frontal, temporal, and parietal lobes (28); abnormal connections are concentrated in the DMN, central executive network, and salience network, while in BD structural changes are concentrated in the frontal lobes, amygdala, and thalamus (29, 30). MDD also shows changes in the frontal lobes, temporal lobes, amygdala and hippocampus, and abnormal connections are concentrated in the DMN, sensorimotor and visual cortex (31, 32). It has also been found that SZ, BD, MDD show co-pathological changes in brain structure and function, and that risk genes for major psychiatric disorders are enriched in the DMN, limbic, and ventral attention networks (33). Differential expression of risk genes is also concentrated in astrocytes and microglia, resulting in patients with altered brain morphology and differences in cortical thickness (34).

In conclusion, the development of multi-scale and multi-omics methods for integrating genetic and medical imaging information provides new insights into the mechanisms and diagnostics of mental illness. These studies conduct in-depth analysis from micro to macro level, multi-scale and multi-model, explore the internal causal relationship between genes, phenotypes and the environment, so as to discover the pathogenesis of diseases, promote the development of targeted therapies, and ultimately achieve disease prediction, diagnosis and precision treatment. The emergence and advancement of high-throughput measurement and more imaging technologies have promoted the development of a new generation of omics research, and the further development of the availability and efficiency of big data has also continuously broken through the dimension of human observation. In the future, researchers should focus more on exploring the transdiagnostic features of major psychiatric disorders to establish a biologically-based disease classification, and developing quantifiable, objective biomarkers that can help provide specific targets for diagnostic and therapeutic interventions.

## Author contributions

L-BC designed the article and guarantor. HY, W-JW and L-BC were responsible for supervision. J-WF wrote the draft and the final version of the manuscript. All authors critically reviewed the report for important intellectual content and approved the final submitted version. All authors had full access to all the data in the study and accept the responsibility to submit it for publication.

## Funding

This work was supported by the Project funded by China Postdoctoral Science Foundation, grant number 2020M683739, the Fourth Military Medical University, grant number 2021JSTS30, Youth Project of General Items for Shaanxi Natural Science Foundation, grant number 2022JQ-908, and National Natural Science Foundation of China, grant number 61976248.

## Conflict of interest

The authors declare that the research was conducted in the absence of any commercial or financial relationships that could be construed as a potential conflict of interest.

## Publisher’s note

All claims expressed in this article are solely those of the authors and do not necessarily represent those of their affiliated organizations, or those of the publisher, the editors and the reviewers. Any product that may be evaluated in this article, or claim that may be made by its manufacturer, is not guaranteed or endorsed by the publisher.
